# Parasite spillover: indirect effects of invasive Burmese pythons

**DOI:** 10.1002/ece3.3557

**Published:** 2017-12-10

**Authors:** Melissa A. Miller, John M. Kinsella, Ray W. Snow, Malorie M. Hayes, Bryan G. Falk, Robert N. Reed, Frank J. Mazzotti, Craig Guyer, Christina M. Romagosa

**Affiliations:** ^1^ Department of Biological Sciences Auburn University Auburn AL USA; ^2^ HelmWest Laboratory Missoula MT USA; ^3^ Everglades National Park National Park Service Homestead FL USA; ^4^ Fort Collins Science Center U.S. Geological Survey Fort Collins CO USA; ^5^ Department of Wildlife Ecology and Conservation Ft. Lauderdale Research and Education Center University of Florida Ft. Lauderdale FL USA; ^6^ Department of Wildlife Ecology and Conservation University of Florida Gainesville FL USA

**Keywords:** biological invasion, Everglades, parasite spillback, parasite spillover, pentastome, *Raillietiella bicaudata*, *Raillietiella orientalis*

## Abstract

Identification of the origin of parasites of nonindigenous species (NIS) can be complex. NIS may introduce parasites from their native range and acquire parasites from within their invaded range. Determination of whether parasites are non‐native or native can be complicated when parasite genera occur within both the NIS’ native range and its introduced range. We explored potential for spillover and spillback of lung parasites infecting Burmese pythons (*Python bivittatus*) in their invasive range (Florida). We collected 498 indigenous snakes of 26 species and 805 Burmese pythons during 2004–2016 and examined them for lung parasites. We used morphology to identify three genera of pentastome parasites, *Raillietiella*, a cosmopolitan form, and *Porocephalus* and *Kiricephalus*, both New World forms. We sequenced these parasites at one mitochondrial and one nuclear locus and showed that each genus is represented by a single species, *R. orientalis*,* P. crotali*, and *K. coarctatus*. Pythons are host to *R. orientalis* and *P. crotali*, but not *K. coarctatus*; native snakes are host to all three species. Sequence data show that pythons introduced *R. orientalis* to North America, where this parasite now infects native snakes. Additionally, our data suggest that pythons are competent hosts to *P. crotali*, a widespread parasite native to North and South America that was previously hypothesized to infect only viperid snakes. Our results indicate invasive Burmese pythons have affected parasite‐host dynamics of native snakes in ways that are consistent with parasite spillover and demonstrate the potential for indirect effects during invasions. Additionally, we show that pythons have acquired a parasite native to their introduced range, which is the initial condition necessary for parasite spillback.

## 1 INTRODUCTION

Direct effects of biological invasions are well documented (e.g., biodiversity loss, predation, and competition; Dorcas et al., 2012; Human & Gordon, 1996; Mooney & Cleland, 2001; Simberloff et al., 2013; Wilcove Rothstein, Dubow, Phillips, & Losos, 1998), but indirect effects, including alteration of host–parasite dynamics, may also profoundly affect an invaded ecosystem (Hoyer et al. 2017; Willson 2017; Dunn et al., 2012 Rogers et al., 2017; Tompkins & Poulin, 2006). Nonindigenous species (NIS) often contain half the parasite species richness of conspecifics in their native range (MacLeod, Paterson, Tompkins, & Duncan, 2010; Torchin, Lafferty, Dobson, McKenzie, & Kurtis, 2003), but still, native hosts are at risk of infection by these non‐native parasites (i.e., spillover; Daszak, Cunningham, & Hyatt, 2000; Tompkins & Poulin, 2006). Adverse effects of NIS‐facilitated spillover may be exacerbated as native hosts do not share a co‐evolutionary history with an introduced parasite and may lack adaptations to effectively counter novel pathogens (Anderson et al., 1986; Daszak et al., 2000; Dogel, Petrushevski, & Polyanski, 1961; Holdich & Reeve, 1991; Kohler & Wiley, 1997).

Nonindigenous species may also acquire parasites from their introduced range (Cornell & Hawkins, 1993; Dobson & May, 1986; Kelly, Paterson, Townsend, Poulin, & Tompkins, 2009a; Poulin & Mouillot, 2003), and in many cases, a majority of parasites infecting NIS are native to the invaded range (Torchin & Mitchell, 2004). If NIS are competent hosts (i.e., parasites are capable of establishment, survival, and reproduction within the host) of native parasites, then NIS may serve as reservoirs of indigenous parasites and increase infection in sympatric native hosts through parasite spillback (Daszak et al., 2000; Kelly et al., 2009a; Tompkins & Poulin, 2006). Aside from direct effects on host mortality, increased transmission can adversely affect competitive interactions and increase vulnerability to predation, resulting in host population declines and altered community structure (Settle & Wilson, 1990; Tompkins, Draycott, & Hudson, 2000; Tompkins, Greenman, & Hudson, 2001). Conversely, if NIS can be infected by parasites from an invaded range, yet the parasites are not able to reproduce within the NIS host, the invading species may act as a sink for native parasites, and subsequently dilute transmission among sympatric native hosts (Kelly, Patterson, Townsend, Poulin, & Tompkins, 2009b; Kopp & Jokela, 2007; Telfer et al., 2005).

Accurate identification of the origin of parasites infecting NIS is vital to understanding mechanisms through which changes to host–parasite relationships occur during biological invasions. Parasite species identification can be challenging if morphological similarities arise from parallel evolution, convergent evolution, or paedomorphism (e.g., Falk, Mahler, & Perkins, 2011; Kelehear, Spratt, Dubey, Brown, & Shine, 2011). Ambiguity of distinguishing characteristics can lead to misidentification and can hinder understanding of how host–parasite relationships are altered during invasions. For example, Barton (1998) used morphological characteristics to identify nematodes from lungs of an invasive Australian population of cane toads (*Rhinella marina*) as *Rhabdias* cf*. hylae*, a species native to Australia. Dubey and Shine (2008) reevaluated Barton's diagnosis using molecular techniques and found the nematodes were, in fact, *Rhabdias pseudosphaerocephala*, a species of South American origin that was likely introduced with cane toads. Dubey and Shine (2008) examined native frogs for the presence of *R. pseudosphaerocephala* and found the introduced parasite was not able to infect native anurans, which presented the opportunity to consider use of *R. pseudosphaerocephala* as a biocontrol tool to manage cane toad populations.

Burmese pythons (*Python bivittatus*) are a large constricting snake species native to Southeast Asia and were introduced to southern Florida through the pet trade. These pythons became established in southern Florida prior to the early 2000s and are now widely distributed (Meshaka, Loftus, & Steiner, 2000; Snow et al., 2007; Willson, Dorcas, & Snow, 2011). Corn, Mertins, Hanson, and Snow (2011) examined ectoparasites of non‐native reptiles in southern Florida and documented new host–parasite relationships among native and introduced ectoparasites for NIS of reptiles, including pythons. Burmese pythons were infested with Neotropical ticks (*Amblyomma rotundatum* and *Amblyomma dissimile*) and two species of chiggers (*Eutrombicula splendens* and *Eutrombicula cinnabaris*) native to the United States. Their results suggest a high degree of host switching among non‐native and native ectoparasites and NIS of reptiles. However, altered host–parasite relationships among endoparasites infecting the invasive python population have not been examined.

**Figure 1:**
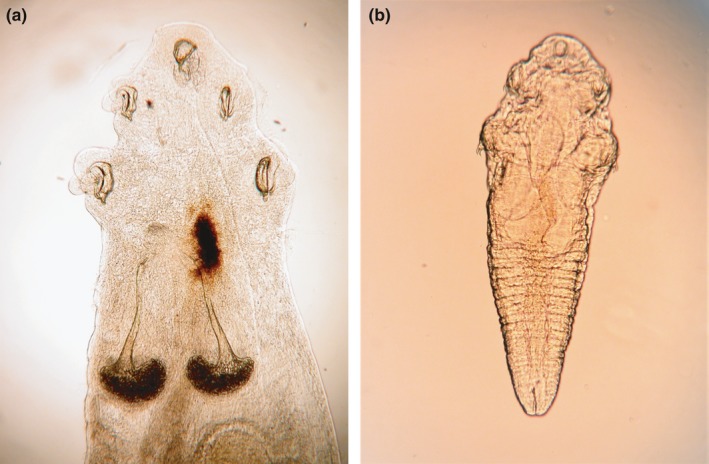
R*aillietiella orientalis* adult (a) and nymph (b) are shown cleared in a phenol solution. *Raillietiella orientalis* is an endoparasite native to Asia that infects the lungs of certain snake species, including Burmese pythons (*Python bivittatus*)

Here, we identified pentastome parasites associated with Burmese pythons in southern Florida as a preliminary step in understanding indirect effects of this invader on the native snake fauna. Despite infecting numerous taxa and being of zoonotic concern, pentastomes remain understudied (Christoffersen & De Assis, 2015). Pentastomes are parasitic arthropods found in the respiratory tract of reptiles, toads, mammals, and birds. However, 90% of definitive hosts are reptiles, with squamates comprising the majority of reptilian hosts (Christoffersen & De Assis, 2013). Pentastomes have an indirect life cycle, often requiring two intermediate hosts. Infective pentastome larvae (nymphs) reside in intermediate hosts (mammals, reptiles, amphibians, fish, and insects), which are consumed by the definitive host. Once ingested, larvae migrate from the definitive host's stomach to its lungs where they complete development and feed on a host's blood (Paré, 2008; Riley, 1986). Pentastome infection, known as pentastomiasis, can induce pathogenic reactions including sepsis and pneumonia that result from decomposed exuviae as a pentastome develops through a series of molts. Scarring, lesions, and hemorrhaging can occur as a parasite migrates through the body and attaches hooks associated with feeding (Jacobson, 2007; Paré, 2008; Riley, 1986). Reduced pulmonary function due to blockage of respiratory passages can occur when a large number of pentastomes infect a host (Riley, 1986). Larval pentastomes can cause adverse reactions within intermediate hosts, with severe visceral infections documented in several mammalian hosts, including humans (Brookins et al., 2009; Self, 1972; Tappe & Büttner, 2009; Tappe et al., 2011). North American pentastomes that infect snake definitive hosts include *Kiricephalus coarctatus*,* Porocephalus crotali*, and *Raillietiella bicaudata*. Colubrid snakes are hosts to *K. coarctatus* and *R. bicaudata*, while *P. crotali* is only known to infect pit vipers. Each of these North American pentastome species has potential to infect invasive Burmese pythons and spillback into native snake populations. Burmese pythons are known hosts to several species of pentastomes in their native range, including *Raillietiella orientalis* (Figure 1), *Armillifer agkistrodontis*,* A. yoshidai*, and *A. moniliformis*, which can be species of zoonotic concern (Christoffersen & De Assis, 2013; Latif, Omar, Chin Heo, Othman, & Tappe, 2011). Burmese pythons may have brought these Asian pentastome species to North America, where spillover into native snake species may occur. Of particular interest is a possibility that two species of *Raillietiella*,* R. bicaudata* and *R. orientalis*, currently occur in southern Florida snakes, where the former evolved in the Americas, and the latter was transported by Burmese pythons from Asia. Thus, examples of spillover and spillback might be present within this single parasite genus.

Because an absence of morphological autapomorphies may impede identification of these pentastome taxa via visual inspection, we employed molecular methods to distinguish among possible species present in our samples. For example, we expected to recover two clades within each genus if two species are present. We used both GenBank and novel sequence data to identify the geographic origin of the samples (i.e., North America or Old World). The presence of Asian pentastomes in native snakes would be a necessary condition for demonstrating spillover, and the presence of North American pentastomes in pythons would be the first necessary condition for demonstrating the potential for parasite spillback.

## 2 METHODS

### 2.1 Snake and pentastome collection

**Figure 2:**
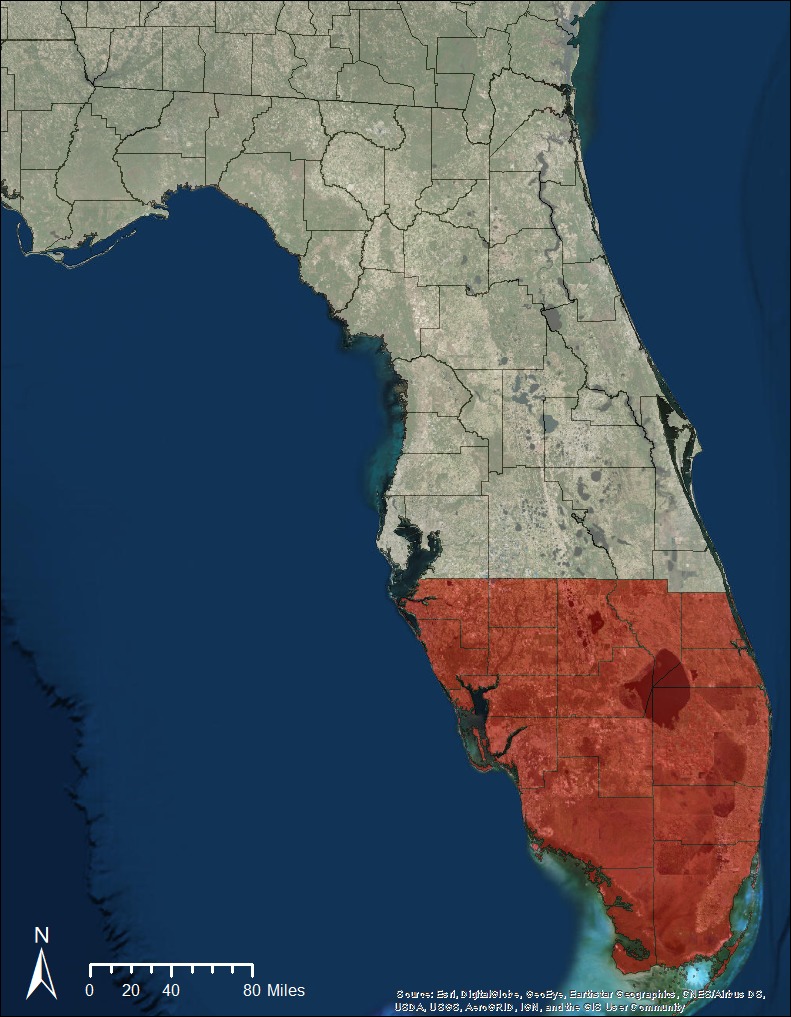
Native snakes were collected from Florida, Georgia, and Alabama from areas of allopatry (gray) with pythons and from Florida in regions of sympatry (red) with pythons

Burmese pythons (*P. bivittatus*;* n* = 805) were collected throughout their introduced range in southern Florida, including Everglades National Park (ENP) and private lands in Collier, Miami‐Dade, and Monroe Counties. These snakes were collected alive or salvaged after being hit by automobiles. Pythons captured alive were euthanized as per Everglades National Park Python Removal Program protocol. We also collected a total of 498 native snakes of 26 species from regions of allopatry (*n* = 228) and sympatry (*n* = 270) with pythons (Figure 2). The allopatric region included Alabama, Georgia, and Florida (north of Hardee, Highlands, Manatee, Okeechobee, and St. Lucie counties). The sympatric region included all remaining counties within Florida. Native snakes from the allopatric region were examined to obtain specimens of *R. bicaudata* from locations that were not confounded by the potential presence of *R. orientalis*. We only collected native snakes that had been previously killed by automobiles to reduce our impact on native snake populations. These snakes were collected during 2012–2016 during nocturnal road surveys conducted on consecutive nights to ensure snakes had been killed within the last 24 hr.

Snakes were dissected immediately after euthanasia or frozen for later dissection. During dissection, the lungs, air sac, oral cavity, trachea, and body cavity were examined for the presence of pentastomes. Areas outside of the lungs and air sacs were examined because pentastomes are known to change location within the body and at times vacate the host entirely when death of the host occurs (Montgomery, Goldberg, Bursey, & Lips, 2006; Paré, 2008). Collected pentastomes were stored in 95% ethanol until morphological and molecular analyses were performed. Examination of morphological traits was performed on pentastomes that were cleared in temporary mounts of 80% phenol and then returned to the preservative. Specimens were identified based on the morphology of the mouth hooks, shape of the mouth opening, and male spicules. Molecular analyses were performed to corroborate our morphological species identification. All pentastomes and snakes collected within National Park Service (NPS) lands were deposited in collections administered by the NPS. All pentastomes and snakes collected outside of NPS lands were deposited at the Auburn University Museum of Natural History.

### 2.2 Molecular methods

We followed the Chelex extraction protocol prescribed by Kelehear et al. (2011) to extract whole genomic DNA from pentastome tissue. Two genes were amplified by polymerase chain reaction (PCR): the mitochondrial gene COI and the nuclear ribosomal RNA gene 18S. Amplification procedures for COI followed Kelehear et al. (2011) using primers LCOI490/HCO2189 (Folmer, Black, Hoeh, Lutz, & Vrijenhoek, 1994). The primers and protocols for 18S amplification followed Brookins et al. (2009). Primers used for PCR amplification were also used for DNA sequencing, which was performed at Beckman Coulter (Danvers, MA). PCR amplification of both COI and 18S was performed on all samples; however, only one gene amplified for some samples. Reactions were performed a second time for failed runs, yet amplification was still not successful for some samples, which were eliminated from the study. Chromatographs from forward and reverse reads were assembled, and contiguous sequences were aligned using Geneious alignment set at default parameters and edited by eye (Geneious version 6.0.6 (http://www.geneious.com, Kearse et al., 2012).

### 2.3 Pentastome phylogeny

Published sequences for COI and 18S available on GenBank were used in combination with sequence data generated from this study to construct a pentastome phylogeny. Published sequences for *Raillietiella* pentastomes included 11 COI sequences from an introduced population of *R. orientalis* collected from Australian snakes (Kelehear, Spratt, O'Meally, & Shine, 2014), one COI sequence of *R. hebitihamata* (*hebitihamata* = *frenata* = *frenatus* and likely = *indica*; Caballero et al., 2015; Kelehear et al., 2011; Poore, 2012; GenBank accession JF975594), one 18S sequence of *R. orientalis* collected in Asia (GenBank accession KC904945), and two 18S sequences labeled as *Raillietiella* sp. (GenBank accession EU370434; AY744887) of Old World origin (Asian and African). Sequence data for *R. bicaudata* were not available from GenBank or from museum collections, and because *R. bicaudata* is reported from snake species that inhabit Florida, Georgia, and Alabama, where we collected our samples, we assumed that any monophyletic lineage that excluded the published *R. orientalis* sequence and exhibited morphology consistent with *R. bicaudata* was that species.

Phylogenetic analyses were performed separately on COI and 18S datasets and then on a concatenated dataset. The concatenated dataset included specimens for which both markers amplified as well as specimens for which only one marker amplified. Sequences for the pentastome *Linguatula arctica* (GenBank accession KF029445.1) were used as an outgroup for the 18S Bayesian and concatenated phylogenies, and *L. serrata* (GenBank accession KU2400601.1) was used as the outgroup for the COI Bayesian phylogeny. Best‐fit models of evolution for the 18S gene, the COI gene, and for the concatenated dataset were selected using Akaike information criteria values in PartitionFinder (Lanfear, Calcott, Ho, & Guindon, 2012). We inferred a Bayesian phylogeny using MrBayes 3.2.2 on CIPRES Science Gateway (Miller, Pfeiffer, & Schwartz, 2010; Ronquist et al., 2012). Each analysis had two runs with four chains each set at default temperatures. These were allowed to run for 10 million generations and were sampled every 1,000 generations. A 25% of burnin was calculated using the sump option, and a 50% majority rule consensus tree was created using the sumt option in MrBayes. Definitive identity and origin of collected pentastomes were determined based upon their position on the tree relative to the position of reference pentastome species.

### 2.4 Haplotype network analyses

Haplotype networks were generated for COI and 18S datasets using PopART (http://popart.otago.ac.nz; Leigh & Bryant, 2015), a program implementing TCS Networks (Clement, Posada, & Crandall, 2000). The TCS Networks were generated using statistical parsimony to estimate the genealogy of the haplotypes present in the dataset using default parameters.

## 3 RESULTS

### 3.1 Pentastome species identification

**Figure 3:**
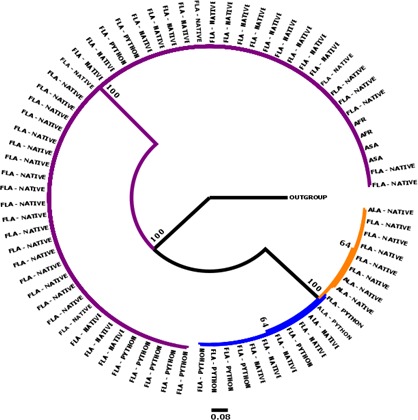
A 50% majority rule Bayesian inference phylogeny inferred from the 18S gene (402 base‐pairs). Clades for *Raillietiella orientalis* (purple), *Porocephalus crotali* (blue), and *Kiricephalus coarctatus* (orange) are shown with collection location of parasite (FLA = Florida; ALA = Alabama; AFR = Africa; and Asia = ASA) and whether the host was native or non‐native (python). Nodes are labeled with the posterior probability as a percent. *Linguatula arctica* was used as an outgroup

**Figure 4:**
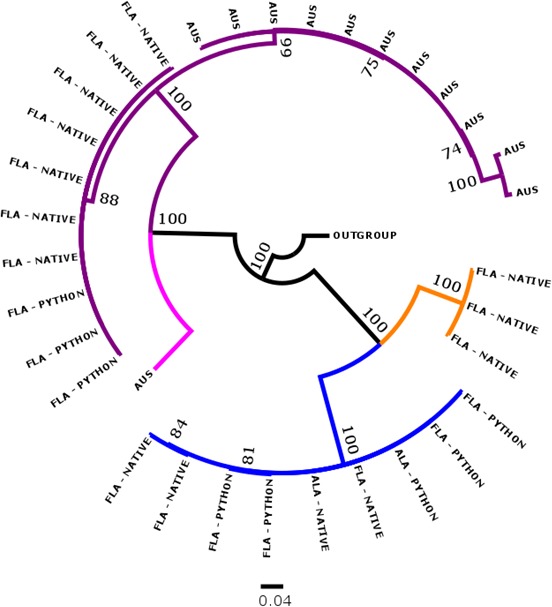
A 50% majority rule Bayesian inference phylogeny inferred from the COI gene (594 base‐pairs). Clades for *Raillietiella orientalis* (purple), *Porocephalus crotali* (blue), *Kiricephalus coarctatus* (orange), and *R. hebitihamata* (pink) are shown with collection location of parasite (FLA = Florida; ALA = Alabama; and Australia = AUS) and whether the host was native or non‐native (python). Nodes are labeled with the posterior probability as a percent. *Linguatula serrata* was used as an outgroup

**Figure 5:**
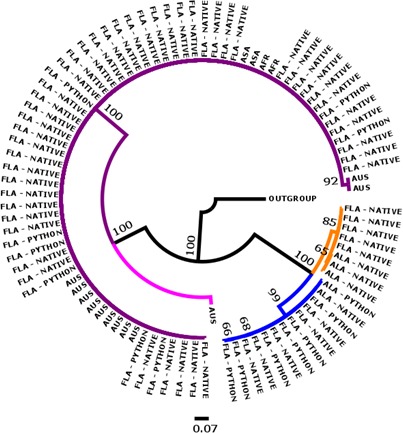
Concatenated tree with a 50% majority rule Bayesian inference phylogeny inferred from the 18S and COI gene (1036 base‐pairs). Clades for *Raillietiella orientalis* (purple), *Porocephalus crotali* (blue), *Kiricephalus coarctatus* (orange), and *R. hebitihamata* (pink) are shown with collection location of parasite (FLA = Florida; ALA = Alabama; AUS = Australia; AFR = Africa; and Asia = ASA) and whether the host was native or non‐native (python). Nodes are labeled with the posterior probability as a percent. *Linguatula arctica* was used as an outgroup

Bayesian analyses of 18S (Figure 3), COI (Figure 4), and concatenation of both genes (Figure 5) resulted in similar topologies. All recovered distinct genetic lineages associated with three genera, *Raillietiella*,* Porocephalus*, and *Kiricephalus. Raillietiella* samples collected from Florida snakes formed a single clade that included the reference specimen from GenBank for *R. orientalis* (18S) and a series of specimens identified as *R. orientalis* from Australian snakes (COI, Kelehear et al., 2014). No Florida specimen of *R. orientalis* was collected from outside the area of sympatry with pythons and phylogenetic reconstructions showed no (18S and concatenated) or limited (COI) structure within the lineage, features consistent with identification of Florida pentastomes as *R. orientalis*. This cluster also included two unidentified *Raillietiella* specimens from GenBank (GenBank accession: EU370434; AY744887) and a larval specimen collected from an African shrew (*Crocidura* sp.) and sent to one of us (JMK) to be identified. In the COI and concatenated trees, we included a specimen of *R. hebitihamata* collected in Australia (GenBank accession: JF975594), the only other member of the genus available from GenBank. This specimen failed to cluster within our Florida specimens, suggesting that other species of *Raillietiella*, if present, would have been revealed on our trees.

Our phylogenetic trees also supported the hypothesis of a single species within each of the other two genera. We identified these genera on morphological grounds as *K. coarctatus* and *P. crotali*, species known from North American snakes. All three phylogenetic analyses recovered these as sister taxa relative to *R. orientalis* (Figures 1, 2, 3). *Porocephalus crotali* was sampled frequently from pythons as well as from native snakes outside the range of pythons. *Kiricephalus coarctatus* specimens from native snakes were recovered from within and outside the range of pythons, but were never collected from Burmese pythons.

### 3.2 Haplotype networks

**Figure 6:**
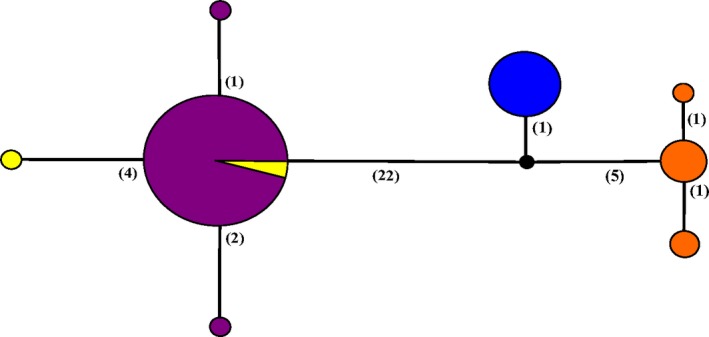
Haplotype network of the 18S gene sequenced from pentastomes collected from Burmese pythons and native snakes is shown. Haplotype variation is shown for *Raillietiella orientalis* (purple = collected from invasive Burmese pythons and sympatric native snakes in Florida; and yellow = Asian/African samples). *Porocephalus crotali* (blue = collected from native snakes sympatric or allopatric with pythons) and *Kiricephalus coarctatus* (orange = collected from native snakes sympatric or allopatric with pythons) are shown for comparison. Each circle represents one haplotype unique to the 18S gene of each group, and the size of the circle corresponds to the number of individuals with that haplotype. Numbers in parentheses represent the number of mutations between haplotypes. Black circles represent hypothetical haplotypes generated by PopART software based upon the 18S gene data provided. Haplotype numbers for each sample are provided as supplemental data

An analysis of the 18S gene revealed that a haplotype of *R. orientalis* obtained in Old World source populations is present in pythons and native snakes from Florida (Figure 6). We documented three additional rare haplotypes, two from Florida and one of Old World origin. We sequenced only a single 18S haplotype from *P. crotali* collected from pythons and native snakes and three haplotypes from *K. coarctatus* collected from native snakes.

**Figure 7:**
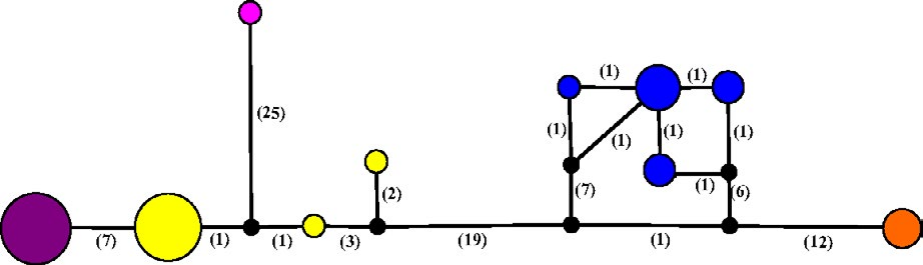
Haplotype network of the COI gene sequenced from pentastomes *Raillietiella*,* Porocephalus*, and *Kiricephalus* collected from Burmese pythons and native snakes. Haplotype variation is shown for *Raillietiella orientalis* collected from invasive Burmese pythons and native snakes in Florida (purple), *R. orientalis* obtained from GenBank from an introduced Australian population (yellow), and *R. hebitihamata* (pink). Haplotypes of *Porocephalus crotali* (blue) and *Kiricephalus coarctatus* (orange) are shown for comparison. Each circle represents one haplotype unique to the COI gene of each group and the size of the circle corresponds to the number of individuals with that haplotype. Numbers in parentheses represent the number of mutations between haplotypes. Black circles represent hypothetical haplotypes generated by PopART software based upon the COI gene data provided. Haplotype numbers for each sample are provided as supplemental data

Haplotype analysis of the COI gene of *R. orientalis* revealed one haplotype from North American samples of pythons and native snakes and three from Australian snakes (Figure 7). No haplotype was shared between *R. orientalis* collected in Florida and those from Australia. Four COI haplotypes were recovered from *P. crotali* and one from *K. coarctatus*.

## 4 DISCUSSION

We provide several lines of evidence that support the hypothesis that the *Raillietiella* samples we collected from both native snakes and invasive Burmese pythons represent a single species, *R. orientalis*, which was introduced to Florida with the Burmese python. First, *Raillietiella* collected from pythons and sympatric native snakes in Florida were contained within a single 18S lineage that included an exemplar of *R. orientalis* collected from an Asian cobra and published to GenBank. Second, the 18S haplotype of this exemplar is shared with specimens from pythons and native snakes of Florida. Finally, specimens of *Raillietiella* are found only in native snakes within the range of invasive pythons, despite extensive sampling of native snakes outside this range. Evidence from the COI gene supports a similar conclusion because of close phylogenetic ties of Florida *Raillietiella* to specimens of *R. orientalis* introduced to Australia.

We suspect Burmese pythons are responsible for the introduction of *R. orientalis* to Florida, as this parasite is known to infect Burmese pythons in their native range of Southeast Asia (Christoffersen & De Assis, 2013). *Raillietiella orientalis* has been noted to infect a wide array of Asian snakes as its primary definitive host, including the widespread snake families Colubridae, Elapidae, and Viperidae, which may result in this parasite being pre‐adapted to infect snakes within its introduced range in the United States and Australia. Florida is home to many introduced reptiles in addition to Burmese pythons (Meshaka, Butterfield, & Hauge, 2004); however, Burmese pythons are the only established snake of Asian origin in Florida that are host of *R. orientalis*. The northern African python is established in Florida (Reed, Krysko, Snow, & Rodda, 2010) and originates from a continent that we determined in this study to contain *R. orientalis*, and this snake is host to pentastomes. However, *R. orientalis* is not known from *P. sebae* in Africa or their introduced Florida population. Moreover, observations of *P. sebae* in Florida are relatively recent and from a limited geographic area, making it less likely that this host transported *R. orientalis* to Florida or plays a significant role in its range expansion.

Our data are the first to document *R. orientalis* from Nearctic snakes. We show that the distribution of this invasive parasite among native snakes is extensive, suggesting that intermediate hosts required for transmission of *R. orientalis* are present in Florida. Intermediate hosts of *R. orientalis* in its native range are unknown, but it has been suggested that raillietiellids have multiple intermediate hosts (Riley, 1986). Kelehear et al. (2014) examined Australian snakes for pentastomes and found 38% of native snakes surveyed were infected with the introduced *R. orientalis*. Due to the similarity among diets of infected snake species, Kelehear et al. (2014) concluded that ground‐dwelling frogs were the likely intermediate host of this pentastome in Australia. Aquatic snakes comprised the majority of native snake species infected with *R. orientalis* in southern Florida, making anurans a plausible intermediate host of this parasite in North America. However, an anuran intermediate host is not likely to allow transmission of *R. orientalis* to invasive pythons. The diet of Burmese pythons in southern Florida is known to include over 40 species, primarily consisting of mammals and birds, occasionally including reptiles (e.g., American alligator, *Alligator mississippiensis*), but not frogs (Dove, Snow, Rochford, & Mazzotti, 2011; Reed & Rodda, 2009; Rochford et al., 2010; Snow et al., 2007). Thus, alternate intermediate hosts must be used by *R. orientalis* in Florida. Our samples include a pentastome nymph collected in Kenya from the mesentery of a shrew (*Crocidura* sp.) and identified as *R. orientalis* by our molecular analysis. This represents the first record of *Raillietiella* from a mammalian intermediate host. Diet overlap of native terrestrial snake hosts and invasive pythons likely includes additional mammalian hosts, especially among rodents.

We searched extensively for *R. bicaudata* and did not recover a lineage attributable to this species in the 498 native snakes examined. Therefore, we question the validity of this taxon. Originally reported from North American colubrid snakes (Heymons, 1935; Heymons & Vitzthum, 1935), the type specimens of *R. bicaudata* include two adult males collected from two captive snakes housed at the Berlin Aquarium in Germany (Christoffersen & De Assis, 2013). These specimens cannot be distinguished from *R. orientalis* or *R. furcocercum* (a species of Central and South American snakes) based on morphological features (J. M. Kinsella, personal observation; Mahon, 1954). Therefore, the type specimens might represent infections of accidental hosts in a zoo environment rather representing a raillietiellid native to North America. The ambiguity surrounding the original description of *R. bicaudata* and its hosts, the lack of morphological traits useful in differentiating pentastomes beyond genus (Riley, 1986), and the fact that we did not recover evidence of *R. bicaudata* in our extensive survey of native snakes lead us to conclude that *R. bicaudata* is not a valid pentastome species. Our example expands the use of molecular data in understanding parasite diversity by noting that these data can be used both to reveal hidden diversity (e.g., Falk et al., 2011; Kelehear et al., 2011) and to reduce apparent diversity.

Our data demonstrate that *P. crotali* is capable of infecting Burmese pythons in Florida. This parasite is native to viperid snakes of North and South America (Riley & Self, 1979), and its presence in invasive Burmese pythons introduces the potential that pythons could negatively impact native pit vipers through parasite spillback (Kelly et al., 2009a). The intermediate hosts of *P. crotali* are primarily small rodents (Christoffersen & De Assis, 2013) including the hispid cotton rat (*Sigmodon hispidus*), Florida mouse (*Peromyscus floridana*), rice rat (*Oryzomys palustris*), and cotton mouse (*Peromyscus gossypinus*) (Kinsella, 1974; Layne, 1967). The diet overlap between pythons and pit vipers aids the transmission of *P. crotali* within these taxa and may result in pythons acting as a reservoir of *P. crotali* infection, with spillback of *P. crotali* from pythons to native pit vipers facilitating increased prevalence of this parasite among pit vipers.

Our observations allowed us to identify pentastomids in invasive pythons and native snakes. We demonstrate that *R. orientalis* has spilled over from pythons to native snakes, an important first step in understanding challenges to conservation of native snake assemblages in southern Florida. The diverse intermediate hosts apparently used by *R. orientalis* have allowed this invasive parasite to infect many native snake species. Several of these native hosts are abundant and widespread, suggesting a fundamental niche for *R. orientalis* that extends well beyond that of the invasive host responsible for the parasite's introduction to Florida. Similarly, the potential of pythons to affect pit vipers through parasite spillback could alter composition of snake predators in an area of intense restoration efforts (Estenoz & Bush, 2015). Our future studies will examine the prevalence, infection intensity, and impact of pentastomes, specifically *R. orientalis* and *P. crotali*, on native snakes and invasive pythons.

## DATA ACCESSIBILITY

Sequence data used to generate haplotype analyses, and 18S, COI, and concatenated phylogenies are available through GenBank (accession numbers MG559564 ‐ MG559658). Data on collection location of parasites and host taxa and haplotype numbers are provided for each parasite sequence utilized in the study as supplemental data.

## CONFLICT OF INTEREST

None declared.

## AUTHOR CONTRIBUTIONS

MAM, RWS, and JMK conceptualized the study. MAM designed the study along with CMR and CG. MAM, RWS, BGF, JMK, RNR, and FJM collected data. MAM performed DNA extractions, PCR, and conducted haplotype analyses. MMH provided guidance on molecular methodologies and performed Bayesian phylogenetic analyses. MAM wrote the original draft of the manuscript. MAM, CG, CMR, MMH, BGF, RNR, JMK, and FJM revised and edited the manuscript.
