# Supplementary material for: Parasite spillover: indirect effects of invasive Burmese pythons
Source: Ecol Evol. 2017 Dec 10;8(2):830–40. doi: 10.1002/ece3.3557 (PMC5773325; doi:10.1002/ece3.3557)
Supplement: Supplementary file 1 [file ECE3-8-830-s001.pdf]

Supplemental Data

Table S1 Catalog number, host species, host identification number, and collection locality are shown for pentastomes (*Raillietiella orientalis*, *Porocephalus crotali* and *Kiricephalus coarctatus)* used in the 18S, COI and concatenated phylogenetic analyses and haplotype analyses. GenBank accession number and haplotype number are provided for each sample for the 18S gene and/or the COI gene. Reference *R. orientalis* samples obtained from GenBank and used in this study are listed in the text with their respective accession number. Haplotype number 1 was obtained from the 18S gene of *R. orientalis* (GenBank accession KC904945) collected in Asia from a Chinese cobra (*Naja atra*). Haplotype number 4 was shared among the 18S gene of Old World samples and *R. orientalis* collected from native Florida snakes and pythons. Parasite catalog number and host ID number are for specimen records at the Auburn University Museum (AUM). Specimens collected within Everglades National Park are retained by that entity; all others are housed at AUM. Host genera are: *P = Python (bivittatus)* and *Pantherophis (guttatus; obsoletus); N = Nerodia; T = Thamnophis; A= Agkistrodon; C = Coluber; D = Drymarchon;* and *L = Lampropeltis).* Samples that were consumed in analyses are noted as NS.

**Parasite Species**	**Parasite Catalog No.**	**Host Species**	**Host ID No.**	**Collection Locality**	**GenBank Accession**	**Haplotype**	
						**COI**	**18S**
P. crotali	28681 A	*P. bivittatus*	1464	Miami-Dade, FL	MG559566		5
P. crotali	28681 A	*P. bivittatus*	1464	Miami-Dade, FL	MG559647	6	
P. crotali	28681 B	*P. bivittatus*	1464	Miami-Dade, FL	MG559567		5
P. crotali	28681 B	*P. bivittatus*	1464	Miami-Dade, FL	MG559652	6	
P. crotali	28722	*P. bivittatus*	914	Miami-Dade, FL	MG559574		5
P. crotali	28722	*P. bivittatus*	914	Miami-Dade, FL	MG559648	7	
P. crotali	28724 A	*A. piscivorus*	23	Covington, AL	MG559578		5
P. crotali	28724 A	*A. piscivorus*	23	Covington, AL	MG559649		9
P. crotali	28724 E	*A. piscivorus*	23	Covington, AL	MG559579		5
P. crotali	28724 E	*A. piscivorus*	23	Covington, AL	MG559650	9	
P. crotali	28688	*P. bivittatus*	718	Miami-Dade, FL	MG559653	8	
P. crotali	28688	*P. bivittatus*	718	Miami-Dade, FL	MG559568		5
P. crotali	28748 A	*A. piscivorus*	53	Alachua, FL	MG559597		5
P. crotali	28748 A	*A. piscivorus*	53	Alachua, FL	MG559654	7	

P. crotali	28749	*A. piscivorus*	55	Alachua, FL	MG559655	7	
P. crotali	28749	*A. piscivorus*	55	Alachua, FL	MG559598		5
P. crotali	41550	*P. bivittatus*	1517	Miami-Dade, FL	MG559607		5
P. crotali	28731 J	*A. piscivorus*	72	Jackson, FL	MG559651	7	
P. crotali	28731 J	*A. piscivorus*	72	Jackson, FL	MG559581		5
P. crotali	41549	*P. bivittatus*	1518	Miami-Dade, FL	MG559631		5
P. crotali	28731 A	*A. piscivorus*	72	Jackson, FL	MG559581		5
P. crotali	28731 B	*A. piscivorus*	72	Jackson, FL	MG559582		5
R. orientalis	28702 A	*P. bivittatus*	862	Miami-Dade, FL	MG559637	1	
R. orientalis	28702 A	*P. bivittatus*	862	Miami-Dade, FL	MG559569		4
R. orientalis	28712 F	*P. bivittatus*	738	Miami-Dade, FL	MG559638	1	
R. orientalis	28712 F	*P. bivittatus*	738	Miami-Dade, FL	MG559571		4
R. orientalis	28712 G	*P. bivittatus*	738	Miami-Dade, FL	MG559639	1	
R. orientalis	28735	*N. fasciata*	339	Miami-Dade, FL	MG559640	1	
R. orientalis	28735	*N. fasciata*	339	Miami-Dade, FL	MG559583		4
R. orientalis	28737	*N. fasciata*	NS	Miami-Dade, FL	MG559584		4
R. orientalis	28746	*N. clarkii*	306	Monroe, FL	MG559596		4
R. orientalis	28746	*N. clarkii*	306	Monroe, FL	MG559641	1	
R. orientalis	28720	*T. sirtalis*	579	Miami-Dade, FL	MG559573		4
R. orientalis	28720	*T. sirtalis*	579	Miami-Dade, FL	MG559643	1	
R. orientalis	28754 A	*A. piscivorus*	98	Miami-Dade, FL	MG559599		4
R. orientalis	28754 A	*A. piscivorus*	98	Miami-Dade, FL	MG559644	1	
R. orientalis	28751 A	*A. piscivorus*	97	Miami-Dade, FL	MG559645	1	
R. orientalis	28752 A	*A. piscivorus*	-	Miami-Dade, FL	MG559642	1	
R. orientalis	28743	*P. guttatus*	458	Monroe, FL	MG559594		4
R. orientalis	28743	*P. guttatus*	458	Monroe, FL	MG559646	1	
R. orientalis	28743 A	*P. guttatus*	458	Monroe, FL	MG559590		4
R. orientalis	28743 B	*P. guttatus*	458	Monroe, FL	MG559591		4
R. orientalis	28743 C	*P. guttatus*	458	Monroe, FL	MG559592		4

R. orientalis	28743 D	*P. guttatus*	458	Monroe, FL	MG559593	4
R. orientalis	41548 A	*P. bivittatus*	1481	Collier, FL	MG559565	4
R. orientalis	41548 B	*P. bivittatus*	1481	Collier, FL	MG559565	4
R. orientalis	28705 B	*P. bivittatus*	743	Miami-Dade, FL	MG559570	4
R. orientalis	28717 A	*T. sirtalis*	578	Miami-Dade, FL	MG559572	4
R. orientalis	28723 A	*N. clarkii*	301	Martin, FL	MG559575	4
R. orientalis	28723 B	*N. clarkii*	301	Martin, FL	MG559576	4
R. orientalis	28723 L	*N. clarkii*	301	Martin, FL	MG559577	4
R. orientalis	28723 F	*N. clarkii*	301	Martin, FL	MG559628	4
R. orientalis	28739 A	*N. fasciata*	344	Miami-Dade, FL	MG559585	4
R. orientalis	28739 B	*N. fasciata*	344	Miami-Dade, FL	MG559586	4
R. orientalis	28739 C	*N. fasciata*	344	Miami-Dade, FL	MG559587	4
R. orientalis	28759 A	*C. constrictor*	164	Miami-Dade, FL	MG559600	4
R. orientalis	28763 A	*C. constrictor*	168	Miami-Dade, FL	MG559601	4
R. orientalis	41551 A	*P. bivittatus*	1516	Miami-Dade, FL	MG559609	4
R. orientalis	41551 B	*P. bivittatus*	1516	Miami-Dade, FL	MG559610	4
R. orientalis	41541 B	*C. constrictor*	*518*	Highlands, FL	MG559613	4
R. orientalis	30269	*C. constrictor*	520	Highlands, FL	MG559614	4
R. orientalis	30087	*C. constrictor*	174	Miami-Dade, FL	MG559615	4
R. orientalis	30055	*C. constrictor*	163	Monroe, FL	MG559620	4
R. orientalis	28734 A	*P. obsoletus*	487	Collier, FL	MG559630	4
R. orientalis	28734 B	*P. obsoletus*	487	Collier, FL	MG559622	2
R. orientalis	28734 C	*P. obsoletus*	487	Collier, FL	MG559621	4
R. orientalis	30280	*N. fasciata*	632	Highlands, FL	MG559625	4
R. orientalis	30271	*P. guttatus*	636	Glades, FL	MG559629	4
R. orientalis	30022 A	*T. sirtalis*	588	Miami-Dade, FL	MG559635	4
R. orientalis	30022 B	*T. sirtalis*	588	Miami-Dade, FL	MG559636	4
R. orientalis	30060	*C. constrictor*	167	Miami-Dade, FL	MG559623	4
R. orientalis	30278 B	*N. fasciata*	359	Glades, FL	MG559602	4
R. orientalis	30278 C	*N. fasciata*	359	Glades, FL	MG559603	4

R. orientalis	30275 A	*N. fasciata*	630	Highlands, FL	MG559604		4
R. orientalis	30275 C	*N. fasciata*	630	Highlands, FL	MG559605		4
R. orientalis	30275 D	*N. fasciata*	630	Highlands, FL	MG559606		4
R. orientalis	41506 B	*A. piscivorus*	65	Collier, FL	MG559612		3
R. orientalis	41506 A	*A. piscivorus*	65	Collier, FL	MG559608		4
R. orientalis	41948	*L. getula*	281	Miami-Dade, FL	MG559619		4
R. orientalis	28736 A	*N. fasciata*	333	Collier, FL	MG559624		4
R. orientalis	30270 A	*D. couperi*	627	Highlands, FL	MG559616		4
R. orientalis	30270 B	*D. couperi*	627	Highlands, FL	MG559617		4
R. orientalis	30278 A	*N. fasciata*	359	Glades, FL	MG559626		4
R. orientalis	30275 B	*N. fasciata*	630	Highlands, FL	MG559627		4
R. orientalis	NS	*Crocidura* sp.	643	Laikipia, Kenya	MG559632		4
K. coarctatus	28725 A	*T. sirtalis*	569	Montgomery, AL	MG559580		8
K. coarctatus	28725 B	*T. sirtalis*	569	Montgomery, AL	MG559633		8
K. coarctatus	28725 C	*T. sirtalis*	569	Montgomery, AL	MG559634		7
K. coarctatus	41525 A	*D. couperi*	628	Highlands, FL	MG559618		6
K. coarctatus	41525 B	*D. couperi*	628	Highlands, FL	MG559611		7
K. coarctatus	28741 A	*N. taxispilota*	303	Miami-Dade, FL	MG559658	10	
K. coarctatus	28740 A	*N. taxispilota*	416	Miami-Dade, FL	MG559656	10	
K. coarctatus	28745	*N. clarkii*	305	Monroe, FL	MG559657	10	
K. coarctatus	28741 A	*N. taxispilota*	303	Miami-Dade, FL	MG559589		7
K. coarctatus	28740 A	*N. taxispilota*	416	Miami-Dade, FL	MG559588		7
K. coarctatus	28745	*N. clarkii*	305	Monroe, FL	MG559595		7

† Species of rat snake (*Pantherophis spiloides* and *P. alleghaniensis*) were designated as *P. obsoletus* complex.
